# Relationship between research activities and individual factors among Japanese nursing researchers during the COVID-19 pandemic

**DOI:** 10.1371/journal.pone.0271001

**Published:** 2022-08-24

**Authors:** Ayano Takeuchi, Shinichiroh Yokota, Ai Tomotaki, Hiroki Fukahori, Yoko Shimpuku, Naoki Yoshinaga

**Affiliations:** 1 Department of Preventive Medicine and Public Health, School of Medicine, Keio University, Tokyo, Japan; 2 Faculty of Medicine, The University of Tokyo, Tokyo, Japan; 3 Division of Informatics, Faculty of Nursing, National College of Nursing, Tokyo, Japan; 4 Division of Gerontological Nursing, Faculty of Nursing and Medical Care, Keio University, Tokyo, Japan; 5 Graduate School of Biomedical and Health Sciences, Hiroshima University, Hiroshima, Japan; 6 School of Nursing, Faculty of Medicine, University of Miyazaki, Miyazaki, Japan; PLOS ONE, UNITED KINGDOM

## Abstract

**Aim:**

To explore the individual factors (such as gender, division of household labor, childcare and elder care) and their impact on research activities in the Japanese nursing research community during the early stage of the COVID-19 pandemic from April to June in 2020.

**Design:**

Cross-sectional study.

**Methods:**

An online survey with a self-reported questionnaire was conducted on Japan Academy of Nursing Science members to explore the impacts of individual factors among Japanese nursing researchers from April to June 2020. A multivariate logistic regression model was used for data analysis.

**Results:**

A total of 1,273 participants (90.7% female, 85.8% university faculty) were included in the analysis. This survey showed that no evidence of a significant gender gap was found in research activities in Japanese nursing researchers during the COVID-19 pandemic. Research activities during the pandemic were associated with time and motivation.

## 1 Introduction

The coronavirus disease 2019 (COVID-19) pandemic from early 2020 has had an impact on academic productivity [1–3]. In particular, researchers have been required to conduct academic and educational activity via telecommuting and the use of information communication technology at home during periods with lockdown or ‘stay-at-home’ orders. In addition, some studies reported that researchers, especially those with roles in childcare and housework, had more time for such activities at home during the pandemic [4–6]. The pandemic has highlighted the challenges that researchers face in both research activity and housework [7]. For example, researchers with at least one child aged under 5 years reported decreasing or unstable productivity compared to those without children or with a child aged 6 years or older [8]. On the other hand, some researchers felt positive changes in their motivation for research activities during the pandemic [2].

The pandemic affected researchers living in Japan where telecommuting and use of information communication technology in education were lagging. After the first case of COVID-19 was confirmed in January 2020, the Government of Japan implemented restrictions in three steps by May 2020 [9], which was around the time of spring vacation and before the beginning of the academic and fiscal years in Japan. The first step was the temporary closure of all Japanese elementary, junior high, and high schools from March 2 to early April 2020. In the second step, the government’s statement requested the cooperation of people living in the special alert area, which included seven regions such as Tokyo and Osaka, to refrain from any non-essential and non-urgent outings and movements, including during the daytime. In the last step, the statement was expanded to the whole country and was later lifted on May 25, 2020. During this period, the education system at universities was changed from face-to-face classes to online teaching [10]. Researchers who were faculty members in universities had to adapt their classes and tutorials to be conducted online, and restructure their own research activities.

Some studies reported that there was a gender gap in research activities due to social and cultural factors during the COVID-19 pandemic [1, 2, 11], but the impact with respect to gender in the academic community in nursing science, which has a high percentage of female researchers, has not yet been reported, as far as we know.

## 2 Background

In Japan, around 90% of graduates with nursing science degrees are women [12]; therefore, most nurses and nursing university faculty members are women. The pandemic had a larger impact on the work of females than males and the situation of females was much more serious than that of males in Japan [13]. On the other hand, jobs in education and health care are being better maintained compared to jobs in other sectors [14]. Clarifying the impact of household childcare and educational effort on research activities during the pandemic will provide important insights for improving the working environment of females in academia [1]. Furthermore, improving the employment environment of the field of nursing, which comprises mostly females, will encourage females to play a more active role in society [15].

Previous studies regarding academic productivity have used bibliometric analysis to focus on the gender gap [4, 8, 16–19] during the COVID-19 pandemic. Such a gender gap is said to reflect social, political, intellectual, cultural, or economic attainment and attitudes [20]. However, the COVID-19 pandemic emphasized the need for more research regarding social and cultural factors in the gender gap.

In order to clarify gender gaps in research activity, it is important to conduct research in the fields in which women’s social advancement is active, and to link personal data such as social and cultural factors and the positive or/and negative impacts on research activities. In addition, exploring factors related not only to negative, but also positive influences on research activities during the pandemic are important for enhancing research activities during a pandemic. To the best of our knowledge, no such study has yet been published. The aims of this study were to demonstrate the relationships between the gender gap and research activities and to explore the impacts of individual factors such as division of household labor, elder care, and childcare in the Japanese nursing research community during the COVID-19 pandemic from April to June in 2020.

## 3 Methods

### 3.1 Design

This was a cross-sectional study

### 3.2 Study setting

This survey was conducted on members of the Japan Academy of Nursing Science (JANS) by the JANS office between July 1 and August 10, 2020 (COVID-19 Nursing Research Countermeasures Committee Member Surveying Team, 2021: NRCCM-ST) [21]. In this survey, NRCCM-ST sent requests for participation to JANS members via email and also posted a request on the JANS website. The current paper was a secondary analysis of academy-led shared research projects [22].

### 3.3 Study participants

All JANS members [22] met the following criteria at the time of applying for membership: (1) a person specializing in nursing science, who is engaged in education or research at a university or college (including junior college), or who practices nursing and has contributed to nursing science, or who has made a research contribution to nursing-related science; and (2) a person with minimum requirements for research achievements for JANS. The total number of JANS members was 9,524 (JANS, n.d.). All JANS members who completed the online survey were included as study participants.

Exclusion criteria were: (1) gender was missing or marked as ‘prefer not to answer’; (2) started a new job, changed jobs, or left a job (retired) from March to June 2020 because this period included the start of the fiscal year, which is a period during which many people change positions. Participants did not receive any incentives.

### 3.4 Methods

The questionnaire included items on demographics, gender, age, academic degree, raising children or not, caring for elderly or other family members, presence or absence of co-resident partner or spouse, affiliations, employment status, presence or absence of full-time employment at a nursing university, working from home or remote work, and receiving a Japanese grant-in-aid for scientific research (KAKENHI). Background information, the materials, methods and aggregated results of this survey were made publicly available [21].

Questions regarding research activities were as follows: the degree of motivation for research activities during the COVID pandemic; the degree of change in the total time spent on research activities; impact of COVID-19 pandemic on overall research activities; percentage allocation of one’s own work time for research, education, management and administration, social contributions, clinical practice and other areas; a questionnaire from ResearchGate asking how the time spent on the ten assessed research activities changed during the pandemic [23]; factors that negatively impacted research activities during the COVID-19 pandemic (negative impacts); and positive changes in research activities during the COVID-19 pandemic (positive influences). ResearchGate is a commercial social networking site for scientists and researchers to share knowledge, answer questions, and find collaborators. Because the ResearchGate survey was the only comparable resource at the time this survey was planned, NRCCM-ST asked the participants to complete the same questionnaire items. Negative impacts included 33 items rated on a six-point scale (not applicable, no impact at all, did not impact much, neither, impacted somewhat, impacted significantly) (‘neither’ is an English translation of the wording of our Japanese questionnaire items and means “neither ‘impacted’ nor ‘not impacted’”). Positive influences included 17 items rated on a five-point scale (not at all, not very much, neither, somewhat, very much).

Excerpts from the survey form are shown in Tables 1 and 2, which list the negative impacts and positive influences, respectively.

**Table 1:** Items on factors that could impact your research activities during the COVID–19 pandemic.

1. Difficulty in in-person contact with study participants
2. Difficulty in entering research facilities/institutions
3. Difficulty in securing means of transport for domestic travel and business trips
4. Difficulty in securing means of transport for overseas travel and business trips
5. Difficulty in accessing equipment, literature, materials, data, computers, and software necessary for research
6. Difficulty in using research technical assistants (including doctoral research assistants)
7. Research efficiency lowered by working from home
8. Difficulty in holding meetings with co-researchers inside/outside your affiliated organization
9. Decreased function of departments, organizations, and institutions related to research (administration, ethics review boards, organizations participating in the research project, partners in outsourcing for surveys and research)
10. Difficulty securing the necessary budget owing to changes to the research plan
11. Difficulty of peer support and communication related to research
12. Slowdown in joint research with co-researchers
13. Slowdown in joint research with graduate students
14. Increase in time for research supervision
15. Delays in the review and publication processes of submitted manuscripts (Japanese/English)
16. Guilt and conflicts in not being able to contribute to COVID-19 measures professionally
17. Increased time spent for lectures (including preparation and assessment)
18. Increased time spent for seminars (including preparation and assessment)
19. Increased time spent for practicum (including preparation and assessment)
20. Increased time spent for clinical practice
21. Increased time spent on the health management of students and staff (e.g., checking health status)
22. Increased time spent on supporting students and staff showing fear of infection
23. Increased time spent on counseling other students and staff (for employment, mental health, economic support)
24. Increased time spent on management/administration (meetings, committee activities, open campus, career workshops)
25. Increased time spent on learning information and communications technology (ICT)
26. Increased time spent on ICT-related support for managers, colleagues, subordinates, and the organization (e.g., installation and support for using online meeting systems)
27. Increased time spent on social contributions related to COVID-19 (e.g., academic society committee activities, public lectures)
28. Increased time spent on housework related to COVID-19
29. Increased time spent on infection prevention and health management related to the effects of COVID-19 in the family
30. Internal and interpersonal conflicts in the family related to COVID-19
31. Increased time spent on childcare owing to COVID19 related closures of daycares, kindergartens, schools, or restricted attendance of school
32. Increased time spent on care of parents or other elderlies related to COVIDstays) 19 (closures of day services and short
33. Guilt and conflicts in not being able to perform COVID19 measures adequately for the housework, childcare, or care for elderlies/parents (e.g., measures to prevent infection in the home)

**Table 2:** Items on positive changes that you might have experienced in your research activities during the COVID–19 pandemic.

1. Found more time for research owing to shortened commute times
2. Found more time for research from adjusting commute times (delayed or earlier commute)
3. Found more time for research from having fewer in-person meetings
4. Found more time for research from canceled or postponed meetings or business trips
5. Built a new lifestyle rhythm
6. Came up with new research ideas
7. Explored and tried new research
8. Increased opportunities to encounter researchers and findings from new areas
9. Came up with ideas for joint research with researchers from new areas
10. Improved the home environment for remote research activities
11. Found more time for research by increasing efficiency for teaching activities remotely
12. Use of ICT increased ease of communication between researchers in Japan
13. Use of ICT increased ease of communication with researchers abroad
14. Increased opportunities for remote research activities
15. Increased opportunities for remote clinical practice
16. Experienced the benefits of remote conferences and workshops
17. Increased opportunities for peer support communication (online casual communication and parties between colleagues or graduate students)

### 3.5 Analysis

At first, we aggregated and confirmed that the participants’ demographics matched the NRCCM-ST (2021) report [21]. Responses for allocation of work time for research, education, management and administration, social contributions, clinical practice and other areas should have totaled 100%. However, some participants made a calculation error and responses that totaled within the range of 90% to 110% were observed; to adjust for calculation errors, the percentages were inverse weighted with degree of error to reach 100%. For example, for participants whose total was 90%, all response percentages were divided by 90 and multiplied by 100. Responses to the questionnaire from ResearchGate (reading how the time spent on the ten assessed research activities changed during the pandemic) were dichotomized as ‘much less’ or ‘less’ (= 1) or ‘others’ (= 0) and totaled. As a result, the total score was distributed from 0 to 10 points. Next, each item evaluating the association between impacts of the COVID-19 pandemic on research activities and negative impacts and positive influences on research was binarized to ‘yes’ or ‘not yes’. ‘Not yes’ included missing data and responses of ‘prefer not to answer’.

Next, to perform factor analysis for shrinking the dimension of items, if any items of the negative impacts and positive influences included missing values or responses of ‘not applicable’, values were assigned as ‘neither’. Exploratory factor analysis for negative impacts and positive influences was used to classify participants as having positive or negative factors, respectively. Promax rotation was used on 33 and 17 items assumed to reflect three hypothetical personality traits for each impact from eigenvalues and scree plots [24]. The factor score of each individual was calculated from the factor loading by the factor analysis, and the group to which each individual belonged was determined.

Univariate and multiple logistic regression models were used to explore the relationship between how much overall research activities were impacted by the COVID-19 pandemic and the related factors. Additionally, subgroup analysis for specific demographics was performed based on the results of regression analyses. Sensitivity analyses for missing data or responses of ‘prefer not to answer’ was conducted in the regression analysis. Free description responses were used as a viewpoint for discussion. Data analysis was performed using SAS (version 9.4).

### 3.6 Ethics

This survey was approved by the institutional review board of the authors’ institution (Approval no. O-0733, June 29, 2020). An explanatory document was presented to the participants online. All participants gave their informed consent online by ticking a checkbox before starting the survey. No confirmation was made as to whether the participants were minors which are those under the age of 20 in Japan or not.

## 4 Results

### 4.1 Participants’ demographics

**Fig 1:**
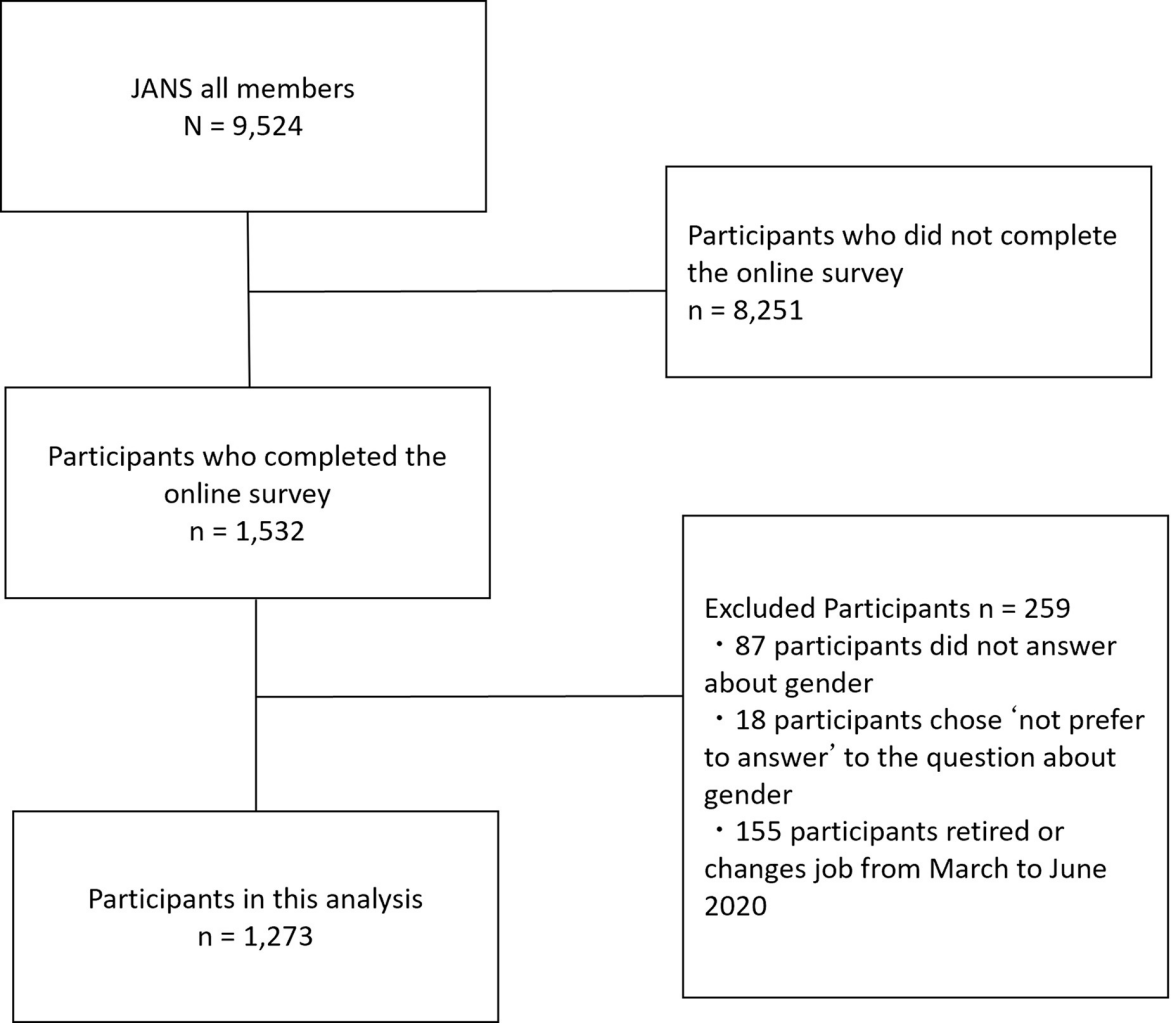
Flow of participants in this study.

A total of 1,532 (16.1%) of 9,524 JANS members completed the online survey, 259 of whom were excluded (87 did not answer, 18 chose ‘not prefer to answer’ to the question about gender, and 155 were retired or changed jobs from March to June 2020), leaving 1,273 members included as participants in this analysis (Fig 1). The participants’ demographics are shown Table 3. Most of the participants were female, university faculty, and employed full time, and about half of them had a Ph.D. Female participants demonstrated the following characteristics more than male participants: over 46 years of age, professor in an academic position, not a graduate student, held a Ph.D., and currently caring for elderly or other family members. Male participants had tendencies to be earlier in their career, an associate professor in an academic position, have a co-resident partner or spouse, and to be raising a child. Table 4 shows the relationship between the participants’ characteristics and how much the COVID-19 pandemic affected their overall research activities.

**Table 3:** Participant characteristics.

			All		Female		Male	
			(n = 1,273)		(n = 1,154)		(n = 119)	
			n	%	n	%	n	%
Demographics								
	Age	<26 years	2	0.16%	1	0.09%	1	0.84%
		26–35 years	92	7.23%	56	4.85%	36	30.25%
		36–45 years	333	26.16%	281	24.35%	52	43.70%
		46–55 years	458	35.98%	439	38.04%	19	15.97%
		56–65 years	324	25.45%	316	27.38%	8	6.72%
		>65 years	42	3.30%	39	3.38%	3	2.52%
		Prefer not to answer	20	1.57%	20	1.73%	0	0.00%
		Unknown	2	0.16%	2	0.17%	0	0.00%
	Main workplace	Nursing university (national)	228	17.91%	197	17.07%	31	26.05%
		Nursing university (prefectural/municipal)	272	21.37%	254	22.01%	18	15.13%
		Nursing university (private)	567	44.54%	521	45.15%	46	38.66%
		University other than nursing university	25	1.96%	21	1.82%	4	3.36%
		Research institute	17	1.34%	5	0.43%	12	10.08%
		Medical, public health, or social welfare institution (e.g. hospitals, clinics, visiting nurse stations)	116	9.11%	112	9.71%	4	3.36%
		Other	20	1.57%	17	1.47%	3	2.52%
		I am not working anywhere/I am not affiliated anywhere	12	0.94%	12	1.04%	0	0.00%
		Prefer not to answer	11	0.86%	11	0.95%	0	0.00%
		Unknown	5	0.39%	4	0.35%	1	0.84%
	Position	Professor	369	28.99%	354	30.68%	15	12.61%
		Associate Professor	235	18.46%	215	18.63%	20	16.81%
		Lecturer	232	18.22%	210	18.20%	22	18.49%
		Assistant professor	212	16.65%	174	15.08%	38	31.93%
		Teaching associate	26	2.04%	23	1.99%	3	2.52%
		Nursing manager	65	5.11%	63	5.46%	2	1.68%
		Clinical nursing professional (full-time)	34	2.67%	28	2.43%	6	5.04%
		Clinical nursing professional (part-time)	17	1.34%	13	1.13%	4	3.36%
		Other	47	3.69%	39	3.38%	8	6.72%
		Prefer not to answer	21	1.65%	21	1.82%	0	0.00%
		Unknown	15	1.18%	14	1.21%	1	0.84%
	Employment type	Full-time with a fixed term	442	34.72%	403	34.92%	39	32.77%
		Full-time with an indefinite term	757	59.47%	689	59.71%	68	57.14%
		Part-time	35	2.75%	28	2.43%	7	5.88%
		Other	14	1.10%	13	1.13%	1	0.84%
		Prefer not to answer	6	0.47%	6	0.52%	0	0.00%
		Unknown	19	1.49%	15	1.30%	4	3.36%
	Graduate student	I am not a graduate student	978	76.83%	899	77.90%	79	66.39%
		Graduate student at a nursing university (Ph.D.)	206	16.18%	179	15.51%	27	22.69%
		Graduate student at a nursing university (Master’s)	18	1.41%	13	1.13%	5	4.20%
		Graduate student at a university program other than nursing (Ph.D.)	40	3.14%	33	2.86%	7	5.88%
		Graduate student at a university program other than nursing (Master’s)	0	0.00%	0	0.00%	0	0.00%
		Other	6	0.47%	5	0.43%	1	0.84%
		Prefer not to answer	3	0.24%	3	0.26%	0	0.00%
		Unknown	22	1.73%	22	1.91%	0	0.00%
	Region of residence (home) designated as the COVID-19 special alert area† from April and to June 2020							
		Yes	809	63.55%	729	63.17%	80	67.23%
		No	449	35.27%	410	35.53%	39	32.77%
		Prefer not to answer	8	0.63%	8	0.69%	0	0.00%
		Unknown	7	0.55%	7	0.61%	0	0.00%
	Highest level of education							
		Ph.D. degree	630	49.49%	589	51.04%	41	34.45%
		Master’s degree	596	46.82%	526	45.58%	70	58.82%
		Bachelor’s degree	29	2.28%	22	1.91%	7	5.88%
		Foundation degree	3	0.24%	2	0.17%	1	0.84%
		Associate degree	1	0.08%	1	0.09%	0	0.00%
		Other	4	0.31%	4	0.35%	0	0.00%
		Prefer not to answer	7	0.55%	7	0.61%	0	0.00%
		Unknown	3	0.24%	3	0.26%	0	0.00%
	Please answer if you selected “1. Ph.D.” Are you a researcher within 8 years of obtaining your Ph.D. degree?							
		Yes	287	45.56%	264	44.82%	23	56.10%
		No	332	52.70%	315	53.48%	17	41.46%
		Prefer not to answer	4	0.63%	4	0.68%	0	0.00%
		Unknown	7	1.11%	6	1.02%	1	2.44%
	Do you currently have a partner or spouse living with you?							
		Yes	757	59.47%	665	57.63%	92	77.31%
		No	433	34.01%	407	35.27%	26	21.85%
		Prefer not to answer	50	3.93%	50	4.33%	0	0.00%
		Unknown	33	2.59%	32	2.77%	1	0.84%
	Are you currently raising children?		0	0.00%				
		Yes	434	34.09%	364	31.54%	70	58.82%
		No	774	60.80%	726	62.91%	48	40.34%
		Prefer not to answer	33	2.59%	33	2.86%	0	0.00%
		Unknown	32	2.51%	31	2.69%	1	0.84%
	Are you currently caring for elderly or other family members?							
		Yes	197	15.48%	187	16.20%	10	8.40%
		No	1011	79.42%	906	78.51%	105	88.24%
		Prefer not to answer	33	2.59%	32	2.77%	1	0.84%
		Unknown	32	2.51%	29	2.51%	3	2.52%
	Are you currently a full-time employee at a nursing university?							
		Yes	1043	81.93%	950	82.32%	93	78.15%
		No	206	16.18%	180	15.60%	26	21.85%
		Unknown	24	1.89%	24	2.08%		
	Has your university implemented working from home or remote work in the previous 3 months (April to June 2020)?							
		Yes, it did	857	67.32%	774	67.07%	83	69.75%
		No, it didn’t	178	13.98%	165	14.30%	13	10.92%
		Others	40	3.14%	39	3.38%	1	0.84%
		Unknown	198	15.55%	176	15.25%	22	18.49%
	Have you received Ministry of Education, Culture, Sports, Science and Technology/Japan Society for the Promotion of Science Grants-in-Aid for Scientific Research (KAKENHI) as the principal investigator for the present fiscal year (FY 2020) (includes continuing research projects started in earlier years)?							
		Yes	582	45.72%	534	46.27%	48	40.34%
		No	666	52.32%	596	51.65%	70	58.82%
		Unknown	25	1.96%	24	2.08%	1	0.84%
Work activities‡								
	How much has your motivation for your research activities changed during the COVID-19 pandemic?							
		Yes (‘much more’ or ‘more’)	1117	87.75%	1023	88.65%	94	78.99%
		Others (‘neither/about the same’, ‘less’, ‘much less’, or ‘unknown’)	156	12.25%	131	11.35%	25	21.01%
	How much has the total time that you spend on your research activities changed during the COVID-19 pandemic?							
		Yes (‘much more’ or ‘more’)	1131	88.85%	1033	89.51%	98	82.35%
		Others (‘neither/about the same’, ‘less’, ‘much less’, or ‘unknown’)	142	11.15%	121	10.49%	21	17.65%
	How much were your overall research activities impacted during the COVID-19 pandemic?							
		Yes (‘much more’ or ‘more’)	221	17.36%	203	17.59%	18	15.13%
		Others (‘neither/about the same’, ‘less’, ‘much less’, or ‘unknown’)	1052	82.64%	951	82.41%	101	84.87%
	How have you allocated your work time in the previous 3 months (April to June 2020) for each item? (%)		Median	Quartile range	Median	Quartile range	Median	Quartile range
		Research (literature search, surveys/experiments, and writing manuscripts/research supervision)	10	(5–20)	10	(5–20)	15	(9–20)
		Teaching (lectures, practicum, seminars)	60	(40–70)	60	(45–70)	50	(40–65)
		Management and administrations (meetings, committees in the university, and open campus days)	20	(10–30)	20	(10–30)	20	(10–30)
		Social contributions (e.g., academic society committee activities, public lectures)	5	(0–10)	5	(0–10)	5	(0–10)
		Clinical practice	0	(0–0)	0	(0–0)	0	(0–0)
		Others	0	(0–0)	0	(0–0)	0	(0–0)

† The COVID–19 special alert area included the following prefectures

Hokkaido, Ibaraki, Tokyo, Kanagawa, Saitama, Chiba, Ishikawa, Gifu, Aichi, Kyoto, Osaka, Hyogo, Fukuoka.

‡ Only full–time faculty members of nursing universities responded to the question item about “work activities” (females n = 950, males n = 93).

**Table 4:** Relationship between the participant characteristics, factors and how much the COVID–19 pandemic affected their overall research activities.

		How much were your overall research activities impacted during the COVID-19 pandemic?			
		Impacted		Others	
		(n = 1,052)		(n = 221)	
		n	%	n	%
How much has your motivation for your research activities changed during the COVID-19 pandemic?	Decreased	579	55.04	39	17.65
How much has the total time that you spend on your research activities changed during the COVID-19 pandemic?	Less	800	76.05	48	21.72
Gender	Female	951	90.40	203	91.86
Age	≤45 years old	347	33.05	80	36.20
Work at a university or not	Yes	907	86.22	167	75.57
Employed full-time or not	Yes	992	94.30	207	93.67
Graduate student or not	Yes	223	21.20	41	18.55
Region of residence (home) designated as the COVID-19 special alert area from April and to June 2020	Yes	668	63.50	141	63.80
Living with a partner or spouse or not	Yes	629	59.79	128	57.92
Raising children or not	Yes	362	34.41	72	32.58
Caring for elderly or other family members or not	Yes	168	15.97	29	13.12
Have your own national grant (KAKENHI) or not	Yes	101	9.60	18	8.14
Negative type 1: Physical factors related to research/time in education		342	32.51	72	32.58
Negative type 2: Mobility limitations in research/communication		397	37.74	31	14.03
Negative type 3: Family matters/mental health condition		313	29.75	118	53.39
Positive type 1: Creating time for research		383	36.41	89	40.27
Positive type 2: Creating opportunities with ICT/increased communication		359	34.13	62	28.05
Positive type 3: New ideas		310	29.47	70	31.67

ICT: information and communication technology.

### 4.2 Factor analysis for negative impacts and positive influences

As a result of factor analysis, negative factors (33 items rated on a six-point scale) were divided into the following three categories: Negative type 1: Physical factors related to research/time in education, Negative type 2: Mobility limitations in research/communication, and Negative type 3: Family matters/mental health condition. Table 5 shows the rotated factor pattern of factor analysis using the 33 items in negative factors. Positive influences (17 items rated on a five-point scale) were also divided into three types: Positive type 1: Creating time for research, Positive type 2: Creating opportunities with information and communication technology (ICT)/increased communication, and Positive type 3: New ideas. Table 6 shows the rotated factor pattern of factor analysis using the 17 items in positive influences.

**Table 5:** Rotated factor pattern of factor analysis using the 33 items in negative factors.

Negative factors	Type 1	Type 2	Type 3
1. Difficulty in in-person contact with study participants	0.043	0.681	-0.080
2. Difficulty in entering research facilities/institutions	0.100	0.696	-0.080
3. Difficulty in securing means of transport for domestic travel and business trips	0.073	0.644	-0.042
4. Difficulty in securing means of transport for overseas travel and business trips	0.056	0.484	-0.023
5. Difficulty in accessing equipment, literature, materials, data, computers, and software necessary for research	0.059	0.372	0.299
6. Difficulty in using research technical assistants (including doctoral research assistants)	0.097	0.465	0.185
7. Research efficiency lowered by working from home	0.156	0.301	0.370
8. Difficulty in holding meetings with co-researchers inside/outside your affiliated organization	0.080	0.616	0.219
9. Decreased function of departments, organizations, and institutions related to research (administration, ethics review boards, organizations participating in the research project, partners in outsourcing for surveys and research)	0.176	0.517	0.306
10. Difficulty securing the necessary budget owing to changes to the research plan	0.149	0.315	0.310
11. Difficulty of peer support and communication related to research	0.129	0.554	0.280
12. Slowdown in joint research with co-researchers	0.204	0.636	0.127
13. Slowdown in joint research with graduate students	0.140	0.448	0.031
14. Increase in time for research supervision	0.239	0.385	0.186
15. Delays in the review and publication processes of submitted manuscripts (Japanese/English)	0.157	0.250	0.206
16. Guilt and conflicts in not being able to contribute to COVID-19 measures professionally	0.135	0.167	0.328
17. Increased time spent for lectures (including preparation and assessment)	0.683	0.221	-0.027
18. Increased time spent for seminars (including preparation and assessment)	0.750	0.157	-0.064
19. Increased time spent for practicum (including preparation and assessment)	0.752	0.122	-0.028
20. Increased time spent for clinical practice	0.340	0.197	0.091
21. Increased time spent on the health management of students and staff (e.g., checking health status)	0.687	0.075	0.235
22. Increased time spent on supporting students and staff showing fear of infection	0.679	0.083	0.205
23. Increased time spent on counseling other students and staff (for employment, mental health, economic support)	0.678	0.041	0.198
24. Increased time spent on management/administration (meetings, committee activities, open campus, career workshops)	0.635	0.136	0.091
25. Increased time spent on learning information and communications technology (ICT)	0.499	0.225	0.186
26. Increased time spent on ICT-related support for managers, colleagues, subordinates, and the organization (e.g., installation and support for using online meeting systems)	0.547	0.147	0.266
27. Increased time spent on social contributions related to COVID-19 (e.g., academic society committee activities, public lectures)	0.128	0.302	0.202
28. Increased time spent on housework related to COVID-19	0.135	0.015	0.772
29. Increased time spent on infection prevention and health management related to the effects of COVID-19 in the family	0.189	0.080	0.750
30. Internal and interpersonal conflicts in the family related to COVID-19	0.055	0.014	0.681
31. Increased time spent on childcare owing to COVID19 related closures of daycares, kindergartens, schools, or restricted attendance of school	-0.048	-0.021	0.591
32. Increased time spent on care of parents or other elderlies related to COVID19	0.089	0.044	0.484
33. Guilt and conflicts in not being able to perform COVID19 measures adequately for the housework, childcare, or care for elderlies/parents	0.126	-0.010	0.694

**Table 6:** Rotated factor pattern of factor analysis using 17 items in positive influences.

Positive influences	Type 1	Type 2	Type 3
1. Found more time for research owing to shortened commute times	0.852	0.133	0.082
2. Found more time for research from adjusting commute times (delayed or earlier commute)	0.848	0.119	0.138
3. Found more time for research from having fewer in-person meetings	0.808	0.203	0.127
4. Found more time for research from canceled or postponed meetings or business trips	0.747	0.196	0.128
5. Built a new lifestyle rhythm	0.598	0.117	0.285
6. Came up with new research ideas	0.240	0.140	0.815
7. Explored and tried new research	0.206	0.144	0.853
8. Increased opportunities to encounter researchers and findings from new areas	0.160	0.282	0.742
9. Came up with ideas for joint research with researchers from new areas	0.161	0.284	0.770
10. Improved the home environment for remote research activities	0.588	0.296	0.265
11. Found more time for research by increasing efficiency for teaching activities remotely	0.539	0.420	0.252
12. Use of ICT increased ease of communication between researchers in Japan	0.228	0.717	0.164
13. Use of ICT increased ease of communication with researchers abroad	0.203	0.675	0.224
14. Increased opportunities for remote research activities	0.269	0.720	0.189
15. Increased opportunities for remote clinical practice	0.153	0.620	0.219
16. Experienced the benefits of remote conferences and workshops	0.040	0.691	0.077
17. Increased opportunities for peer support communication (online casual communication and parties between colleagues or graduate students)	0.155	0.687	0.111

The factor score for each participant determined which positive type and negative types they belonged to (e.g., one participant could belong to Positive type 1 and Negative type 3).

### 4.3 Association between positive and negative factors and participant characteristics

In Table 7, the associations between positive and negative types and participants’ characteristics were aggregated. Fewer participants belonging to “Negative type 3 (Family matters/mental condition)” indicated that their research activities were inhibited, motivation was decreased, and time spent on research decreased. In addition, graduate students were not able to acquire new opportunities for research.

**Table 7:** Association between positive and negative factors and participant characteristics.

		Negative type						Positive type					
		1: Physical factors related to research/time in education		2: Mobility limitations in research/communication		3: Family matters/mental condition		1: Creating time for research		2: Creating opportunities with ICT/increased communication		3: New ideas	
		(n = 414)		(n = 428)		(n = 431)		(n = 472)		(n = 421)		(n = 380)	
		n	%	n	%	n	%	n	%	n	%	n	%
How much were your overall research activities impacted during the COVID-19 pandemic?	Impacted	342	82.61	397	92.76	313	72.62	383	81.14	359	85.27	310	81.58
How much has your motivation to your research activities changed during the COVID-19 pandemic?	Decreased	206	49.76	229	53.50	183	42.46	225	47.67	207	49.17	186	48.95
How much has the total time that you spend on your research activities changed during the COVID-19 pandemic?	Less	311	75.12	311	72.66	226	52.44	279	59.11	303	71.97	266	70.00
Gender	Female	377	91.06	391	91.36	386	89.56	419	88.77	385	91.45	350	92.11
Age	<45 years old	133	32.13	111	26.06	183	42.46	168	35.59	152	36.19	107	28.23
Work at university or not	Yes	388	93.72	379	88.55	307	71.23	382	80.93	376	89.31	316	83.16
Employed full-time or not	Yes	408	98.55	408	95.33	383	88.86	428	90.68	407	96.67	364	95.79
Graduate student or not	Yes	82	19.81	78	18.22	104	24.13	129	27.33	85	20.19	50	13.16
Region of residence (home) designated as the COVID-19 special alert area from April and to June 2020	Yes	248	59.90	275	64.25	286	66.36	316	66.95	264	62.71	229	60.26
Living with a partner of spouse or not	Yes	243	58.70	231	53.97	283	65.66	290	61.44	248	58.91	219	57.63
Raising children or not	Yes	133	32.13	96	22.43	205	47.56	165	34.96	158	37.53	111	29.21
Caring for elderly or other family members or not	Yes	70	16.91	53	12.38	74	17.17	72	15.25	52	12.35	73	19.21
Have your own national grant (KAKENHI) or not	Yes	192	46.38	251	58.64	139	32.25	211	44.70	213	50.59	158	41.58
		%	SD	%	SD	%	SD	%	SD	%	SD	%	SD
How have you allocated your work time in the previous 3 months (April to June 2020) for research? (%)		11.33	9.50	15.68	11.64	16.70	14.27	16.22	13.55	13.99	11.45	12.74	10.30
How have you allocated your work time in the previous 3 months (April to June 2020) for teaching? (%)		58.97	18.08	56.68	17.39	54.37	22.86	56.39	19.12	56.17	19.94	58.12	19.26

ICT: information and communication technology; SD: standard deviation.

### 4.4 Impacts on research activities

Table 8 shows the results of logistic multiple regression with the outcome of whether or not research activities were inhibited (based on binarized responses). Decrease in motivation, decrease in time available, and lower number of ResearchGate items influenced the inhibition of research. By types of factor analysis, the odds ratio of “Negative type 1 (Physical factors related to research/time in education)” was 3.55 times (95% confidence interval [CI]: 2.15–5.5) higher than “Negative type3 (Family matters/mental health condition)”. The results showed that “Negative type2 (Mobility limitation in research/communication)” was 3.13 times (95%CI: 1.88–5.24) more likely than “Negative type1 (Physical factors in research/time in education)” to feel that their research was significantly inhibited. Male participants were 1.67 times (95%CI: 0.89–3.16) likely to be inhibited than female participants. Living with a partner or spouse, raising children or not, and caring for the elderly or other family members were not significant, but those with family burden tended to feel more inhibited. The results of the sensitivity analysis were similar when missing values and responses of “prefer not to answer” were included.

**Table 8:** Multivariate logistic regression results for the relationship between how much overall research activities were impacted during the COVID–19 pandemic and the related factors.

		OR	95%CI
How much has your motivation to your research activities changed during the COVID-19 pandemic?	Decreased/not	1.98	(1.27–3.07)
How has the time you spend on the following activities changed during the COVID-19 pandemic?	Decreased/not	3.39	(2.19–5.24)
ResearchGate items	/10 points	1.55	(1.40–1.73)
Negative impacts	Type 1[Physical factors in research/time in education] / Type 3[Family matters/mental health]	1.13	(0.74–1.74)
	Type 2[Mobility limitations in research/communication] / Type 3[Family matters/mental health]	3.55	(2.15–5.87)
	Type 2[Mobility limitations in research/communication] / Type 1[Physical factors in research/time in education]	3.13	(1.88–5.24)
Positive influences	Type 1[Creating time for research] / Type 3[New ideas]	1.21	(0.78–1.88)
	Type 2[Creating opportunities with ICT/increased communication] / Type 3[New ideas]	1.48	(0.93–2.36)
	Type 2[Creating opportunities with ICT/increased communication] / Type 1[Creating time for research]	1.23	(0.79–1.91)
Gender	Male/female	1.67	(0.89–3.16)
Age	≤45 years/>45 years	0.92	(0.61–1.40)
Work at university	Yes/not yes	1.37	(0.84–2.24)
Employed full-time	Yes/not yes	0.48	(0.22–1.04)
Graduate student	Yes/not yes	1.37	(0.86–2.18)
Region of residence designated as the COVID-19 special alert area	Yes/not yes	0.87	(0.60–1.27)
Living with a partner or spouse	Yes/not yes	1.08	(0.72–1.62)
Raising children or not	Yes/not yes	1.24	(0.80–1.91)
Caring for elderly or other family members or not	Yes/not yes	1.37	(0.80–2.35)

OR: odds ratio; 95%CI: 95% confidence interval; ICT: information and communication technology.

## 5 Discussion

The target participants of this study were 90.7% female and 9.3% male (Table 3). According to the authors’ tabulation from the results of the 2019 Basic School Survey by the Ministry of Education, Culture, Sports, Science and Technology of Japan [12], the gender composition of faculty members at four-year universities and junior colleges in Japan is 26.4% female and 73.6% male. The gender ratio in the target participants of this study was different from that of faculty members overall in Japan, and the proportion of females was overwhelmingly large. According to the Report on Public Health Administration and Services (Practicing health professionals) by the Ministry of Health, Labour and Welfare [25], employed public health nurses, midwives, nurses, and practical nurses are comprised of 92.7% females and 7.3% males. Therefore, the gender ratio of the target participants in this study reflects the gender ratio of employed nursing professionals in Japan.

According to the Report on Public Health Administration and Services, the number of men in the nursing profession is increasing every year. Because nursing was originally a predominantly female occupation, there were more females than males in our study group, most were 46 years of age or older, there were more females who are currently professors, more with doctoral degrees than graduate degrees, and more were elderly and had families to support. The following characteristics of the male study participants were also observed: a tendency to be younger, to have more children to care for, and to be less likely in professorial positions. Other demographic characteristics, including education and job title, are biased toward women in general because the participants are nursing researchers, but it should be noted that JANS is the largest society of nursing researchers in Japan.

As shown in Table 4, more researchers who answered that their research activities were inhibited felt that their motivation for research had decreased and also felt that their research time had decreased compared to those who answered that their research activities were not inhibited. University faculty members were more likely to say their activities were inhibited and to have negative impacts related to research activities. This difference suggests that faculty members at universities, who were in a position to combine research with teaching (including clinical internship), may have felt that their research was impeded by the time taken for teaching. In Japan, it is known that university faculty members are in charge of clinical internships and have a heavy preparation burden [26]. It is possible that this burden was further increased by the COVID-19 pandemic. On the other hand, those who reported being inhibited were less likely to have negative factors related to family matters than those who reported not being inhibited. There was no difference between those who said they were inhibited and those who said they were not in the following questions: gender; age; whether or not they were residents of special alert areas; whether or not they had family living with them; whether or not they had children; whether or not they were caregivers; and whether or not they had positive influences. The details will be interpreted in conjunction with the multivariate results in Table 8.

As seen in Table 7, those who belonged to “Negative type 3 (Family matters/mental condition)” (72.6%), tended to answer that their research was not inhibited more than those who belonged to “Negative type 2 (Mobility limitations in research/communication)” (92.8%). Similarly, those who belonged to “Negative type 3” (42.5%) were less likely to say that they were less motivated than those who belonged to “Negative type 2” (53.5%). This may reflect a cultural characteristic of Japanese people in which they tend to be patient, and originating from the ancient Buddhist belief that they are responsible for their own work-related disruptions due to family factors or other private circumstances, and that they are reluctant to answer that they have been hindered or that their motivation has decreased. Those who belonged to “Negative type 3” had higher percentages of co-residence and providing childcare than those whose research was inhibited (65.7% and 54.0%, 47.6% and 22.4%, respectively). It was suggested that the disruption of research activities due to cohabitation and childcare may not have been viewed as ‘disruption’ by participants when answering the questionnaire.

Fewer graduate students (13.2%) felt that the COVID-19 disaster provided them with new opportunities, while a higher percentage of graduate students (27.3%) felt that it provided them research time. In other words, compared to researchers who were already independent, graduate students, who are still receiving guidance in their research activities, may have generated more research time, but this did not lead to new opportunities. It should be noted that many of the respondents (14.69%) were working as faculty members while enrolled in doctoral programs. Since such people had to conduct research in a limited period of time, they may be more likely to feel that their research has been hampered more severely by the COVID-19 pandemic.

Based on the results of multiple logistic regression analysis, Table 8 shows that motivation, time, and ResearchGate items clearly influenced the inhibition of research, indicating that there were some differences, unlike in previous studies in which males tended to be more inhibited than females (odds ratio: 1.67, 95%CI: 0.89–3.16) [1, 2, 11]. This may be because males in this study were more likely than females to be younger, to be living together with family, and to have children. Although we could not conclude that there is a gender gap, this study showed that those who reported having a heavy family burden also experienced inhibited research, indicating a need for improvements in the work environment regardless of gender.

Items related to living with a partner or spouse, raising children or not and caring for elderly or other family members or not tended to inhibit research, but were not statistically significant. This survey did not include a mental health questionnaire, as in the previous study by Liu 2021 [27]. Since this survey was conducted in the early stages of the pandemic and at a time when there was no large-scale lockdown in Asia except for in China [28], it is possible that the impact of the pandemic had not yet manifested itself. We will continue to conduct ongoing surveys. There was no statistically significant difference in whether or not a participant lived in a specific warning area. In the spring of 2020, the period covered by the survey, changes in the educational environment and various demands for activity restrictions across Japan may have had an impact, even if individuals did not live in the specific prefectures that were affected. Since this survey was a recall type, there may be a recall bias in that some participants may have provided responses for the time when the survey was actually conducted (July to August 2020) instead of the target period (April to June 2020, during the state of emergency), because warning information was still being issued in each prefecture even after the state of emergency ended.

The participants’ research efforts were reduced due to childcare and nursing care. It is important to make it easier for nurses to take leave to care for their children and to develop social infrastructure such as nursery schools and elder care and welfare facilities, even under special restrictions such as during disasters or a pandemic. While it would be ideal from an individual perspective to prepare society as a whole in advance for an unprecedented pandemic, it is not realistic from a financial perspective. On the other hand, there were some respondents who felt positive influences, such as being able to carry out their research in a remote work environment at home, and discovering new opportunities. Of course, there are many areas of research that cannot be conducted remotely, such as in vitro and in vivo research. Depending on the research area, it was necessary to have already been familiar with and to have used technologies such as remote conferencing for interviews and web-based questionnaires in normal times before the pandemic.

### Limitations and future challenges

Because this survey was completed entirely online and only aggregated data that did not personally identify individuals was used, the possibility of participants being identified to employers or funding sources is unlikely to have skewed responses. However, this study does have some limitations. First, as the study design was a cross-sectional survey, the causal association between motivation and gender was not examined. Second, this questionnaire was originally created for this project and has not been sufficiently verified for reliability and validity, partly because it was created by Corona Peripherals with an emphasis on speed. Among the many items, we chose to use simple indicators such as increase or decrease in burden and objective indicators such as increase or decrease in time as much as possible for the analysis. Third, normally, the results would have been obtained by adjusting for confounding as a covariate, but in this case, considering the possibility of missing information, we included the response ‘prefer not to answer’ and missing data into the ‘not yes’ category. Therefore, there is a possibility of residual confounding. Finally, the study population was dominated by full-time and tenure-track workers, possibly due to the large number of mid-career to senior researchers who are conducting nursing research. This bias of the participants limited on extrapolation of this study. Furthermore, the response rate from JANS members was low. This study has a potential selection bias.

## 6 Conclusion

This survey showed that no evidence of a significant gender gap was found in research activities in Japanese nursing researchers during the COVID-19 pandemic in Japan. The participants who belonged to “Negative type 3 (Family matters/mental condition)” or “Negative type 1 (Physical factors related to research/time in education)” were more inhibited than who belonged to “Negative type 2 (Mobility limitations in research/communication)”. In addition, it is important for research to be familiar with the most current telecommunication-related solutions.
